# Determination of nitrofuran metabolites in sausage casings and crawfish using LC-Q-Orbitrap HRMS: Method development and validation

**DOI:** 10.1016/j.toxrep.2025.102076

**Published:** 2025-06-19

**Authors:** Omar Khaled, Lamia Ryad, Nermine Gad, Fawzy Eissa

**Affiliations:** a Agriculture Research Centre, Central Laboratory of Residue Analysis of Pesticides and Heavy Metals in Foods, Ministry of Agriculture, Giza 12311, Egypt; b Environment and Bio-Agriculture Department, Faculty of Agriculture, Al-Azhar University, Nasr, Cairo 11884, Egypt

**Keywords:** Nitrofuran metabolites, Residue determination, Food safety, AHD, AMOZ, AOZ, SEM

## Abstract

This study developed and validated an analytical method for determining four nitrofuran (NF) metabolites in sausage casings and crawfish matrices. The method utilizes liquid chromatography coupled with quadrupole-Orbitrap high-resolution mass spectrometry (LC-Q-Orbitrap HRMS) to analyze these metabolites. The validation of the developed method was carried out in accordance with the Commission Implementing Regulation (CIR) EU 2021/808, using three concentration levels ranging from 0.25 to 0.75 μg/kg. The recoveries ranged from 77 % to 109 %, while both repeatability and reproducibility remained consistently below 15 %. The calibration curves exhibited good linearity, with correlation coefficients (R) exceeding 0.9978. The limits of detection (LOD) ranged from 0.0218 to 0.0596 μg/kg, while the limits of quantification (LOQ) ranged from 0.0719 to 0.1966 μg/kg. The decision limit (CCα) and detection capability (CCβ) values ranged from 0.30 to 0.39 μg/kg and 0.29–0.35 μg/kg, respectively. Out of 30 crawfish samples collected from local markets in Egypt, 16.66 % contained NF metabolites residues, whereas sausage casings showed no NF metabolite residues. The reliability of the method was further demonstrated through successful participation in two proficiency testing (PT) rounds.

## Highlights

• A method for detecting NF metabolites in sausage casings and crawfish was developed.
• The analytical method was fully validated in accordance with CIR EU 2021/808.
• LOD and LOQ ranged from 0.0218–0.0596 µg/kg and 0.0719–0.1966 µg/kg, respectively.
• 16.66 % of crawfish samples had nitrofuran residues, while sausage casings had none.

## Graphical Abstract

**Figure:**
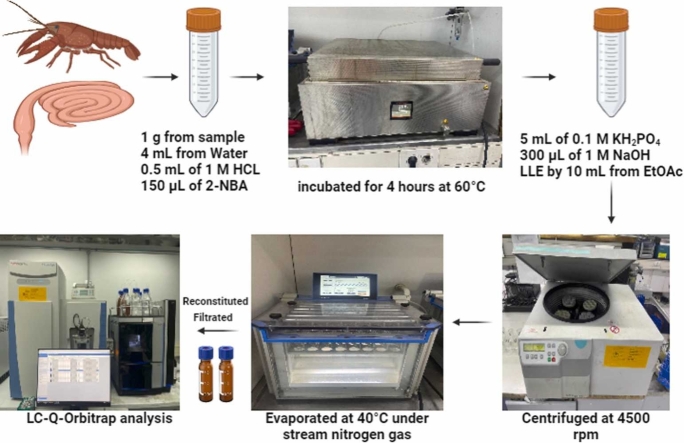

## 1 Introduction

Nitrofurans (NFs) are a class of broad-spectrum antibiotics extensively used to inhibit or eliminate bacterial growth. They are effective against both gram-negative and gram-positive bacteria, as well as fungi and protozoa [1], [2]. NF antibiotics have found widespread application across various sectors of animal husbandry, including poultry, swine, bovine, and aquaculture industries, particularly in the farming of fish and crustaceans [3], [4]. Due to their cost-effectiveness, accessibility, and potent efficacy against drug-resistant pathogens, NFs were extensively employed in the livestock and aquaculture industries as prophylactic and therapeutic agents [5]. The administration of NFs, including furaltadone (FT), furazolidone (FZ), nitrofurazone (NZ), and nitrofurantoin (NT), initiates metabolic processes that generate side-chain metabolites. These metabolites, namely 3-amino-5-morpholinomethyl-2-oxazolidinone (AMOZ), 3-amino-2-oxazolidinone (AOZ), semicarbazide (SEM), and 1-aminohydantoin (AHD), serve as respective marker residues for the detection of FT, FZ, NZ, and NT [6], [7], [8]. The side-chain metabolites of NF antibiotics can persist in animal tissues for extended periods, ranging from several weeks to potentially months, as they form stable adducts with cellular proteins, resulting in the accumulation of protein-bound residues [9]. This long-term persistence of NF metabolites in animal tissues increases the risk of exposure through the consumption of contaminated animal products. Owing to their carcinogenic and mutagenic properties, both the parent NF compounds, and their metabolites have been prohibited for use in food-producing animals across most regulatory jurisdictions worldwide [10], [11]. Their metabolites have the potential to disrupt the process of DNA replication in both bacterial and mammalian cells, leading to the suppression of DNA synthesis [12]. Consequently, NFs and metabolites have been incorporated into Annex IV of the European Commission Regulation 1442/95, which explicitly bans their application in the livestock and poultry industries due to the associated public health risks [13]. The European Commission has recently established a Reference Point of Action (RPA) of 0.5 μg/kg for NF metabolites in food products derived from animals [14]. Despite being banned in several major jurisdictions, including the European Union, the United States, and China, NF antibiotics continue to be utilized due to their effectiveness, low cost, and accessibility through online purchases, which circumvent regulatory restrictions [15]. This ongoing use poses a significant risk to human and animal health, as it bypasses regulatory restrictions designed to protect consumers. Eissa & Younes. [16] reported that, according to the Rapid Alert System for Food and Feed (RASFF) notifications regarding fish from 2000 to 2022, NF metabolites were detected in fish samples 25.85 % of all detected residues of veterinary medicinal products, with AOZ and SEM concentrations ranging from 0.39 to 140 μg/kg and from 0.53 to 122 μg/kg, respectively. The detection of metabolites derived from veterinary drugs in animal-derived food and aquaculture products poses a significant challenge to international trade, as it not only escalates the likelihood of trade disputes between importing and exporting nations but also constitutes a crucial aspect of technical trade barriers among countries. It is important to acknowledge that NFs continue to be approved for use in human medical applications and are widely available through international markets [10]. Consequently, there is a pressing need to develop and implement sensitive analytical methods capable of detecting and quantifying these compounds at trace levels to ensure regulatory compliance and protect public health. The analysis of NF metabolites in food is challenging due to their binding with proteins, high hydrophilicity, and low molecular weight. These characteristics hinder isolation, extraction, chromatographic separation, and detection. Acid hydrolysis using hydrochloric acid at moderate temperatures for prolonged periods is essential to isolate these associated metabolites. Additionally, derivatization with 2-nitrobenzaldehyde (2-NBA) is necessary to enhance detection sensitivity [4], [17], [18]. Enzyme-linked immunosorbent assay (ELISA) based methods have been extensively employed as a screening approach for the determination of NF metabolites, offering a valuable alternative to chromatographic techniques due to their ability to detect small molecules effectively and efficiently [19], [20], [21], [22]. One limitation of employing ELISA for the simultaneous analysis of all four NF metabolites is the necessity to use four distinct assay plates, which can be time-consuming and resource-intensive, potentially hindering the efficiency of the testing process [23]. Consequently, the specialized instrumentation necessary for these analytical techniques render them impractical for field-based applications and the concurrent detection of multiple contaminants, limiting their utility in certain scenarios where rapid, on-site testing is required. In the past, several analytical approaches for the determination of NF metabolites were described, which employed high performance liquid chromatography coupled with either ultraviolet (HPLC-UV) detection or UV photodiode array detection, as reported by Conneely et al. [24]. More recently, the use of HPLC Diode Array Detection (HPLC-DAD) [25], liquid chromatography in combination with tandem mass spectrometry (LCMS/MS) has gained prominence as a powerful analytical tool for the identification and quantification of NF metabolites [18], [26], [27], [28]. In the context of ensuring food safety through the application of mass spectrometry techniques, Orbitrap mass spectrometry stands out as a superior choice compared to UPLC-MS/MS [29]. However, most studies focus on NF metabolites detection in fish and shrimp [25], [30], [31], [32], [33]. Studies analyzing NF metabolites in crawfish, a popular crustacean, are scarce as well as in sausage casings remains limited. This study aims to address these gaps by developing and validating a sensitive and reliable liquid chromatography-quadrupole-Orbitrap high-resolution mass spectrometry (LC-Q-Orbitrap-HRMS) method for the simultaneous determination of NF metabolites in sausage casings and crawfish using liquid liquid extraction (LLE). This method will contribute valuable data for regulatory monitoring of these specific food products sourced from the Egyptian market.

The findings of this investigation provide valuable insights into the potential health risks associated with the presence of NF metabolite residues in these food products. The detection of these residues raises significant concerns about consumer well-being, especially considering the limited number of studies that have been conducted in this particular field within the Egyptian context. By shedding light on this issue, our research contributes to a better understanding of the current situation and underscores the need for further investigations and interventions to safeguard public health and maintain food safety standards in Egypt.

## 2 Materials and methods

### 2.1 Chemicals and reagents

All the analytical standards of NF metabolites utilized in this work, including 3-amino-5-morpholinomethyl-2-oxazolidinone (AMOZ), 3-amino-2-oxazolidinone (AOZ), 1-aminohydantoin hydrochloride (AHD), and semicarbazide hydrochloride (SEM), were obtained from Dr. Ehrenstorfer (Augsburg, Germany) and were of high purity (≥98 %). LC-MS grade acetonitrile (ACN), methanol (MeOH), anhydrous di-potassium hydrogen phosphate (K_2_HPO_4_), and 2-nitrobenzaldehyde (2-NBA) were purchased from Merck (Darmstadt, Germany). Hydrochloric acid (HCl) (≥37 %), Ammonium formate, Pure formic acid (99 %), and sodium hydroxide (NaOH) (≥99.8 %) were obtained from Sigma-Aldrich (Darmstadt, Germany). The ethyl acetate (EtOAc) (≥98 %) was purchased from Honeywell (Charlotte, North Carolina, United States). The acquisition of ultra-pure water was accomplished by the utilization of a MilliQ UF-Plus system, manufactured by Millipore in Germany. Individual compound stock solutions were prepared at a concentration of 1000 μg/mL in MeOH, stored in glass containers, and kept at −18 °C for one year, which is within their stability period. The blended working standard solution of 10 μg/L utilized in the study was diluted from the stock solutions and stored at 4 °C for three months. To prepare a 50 mM solution, 2-NBA was dissolved in MeOH. The dissolving solution was prepared by mixing 48 mL of MeOH, 20 mL of 50 mM ammonium formate, and 32 mL of water to obtain a total volume of 100 mL.

### 2.2 Apparatus

The centrifuge was obtained from Hermle (Gosheim, Germany). The nitrogen gas evaporator (Turbovap LV) was supplied by Biotage (Danmarksgatan, Sweden). A water bath with thermostat was sourced from Memmert (Schwabach, Germany). The pH meter was obtained from Mettler Toledo (Greifensee, Switzerland) and calibrated before use with certified calibration standards of pH 4, 7, and 10.

### 2.3 Sample collection

Thirty-five randomly selected samples, comprising sausage casings and red swamp crawfish (Procambarus clarkii), were procured from multiple local markets throughout Egypt to ensure a representative sampling of consumer goods. To reduce selection bias, the samples were chosen at random from different sources. Sterile equipment and containers were employed during the collection process. Following collection, the samples were unambiguously labeled and promptly chilled in an insulated container for same-day transport to the laboratory. Upon receipt, the samples were stored at a temperature of −20°C to maintain their integrity until the commencement of the analysis. The blank samples used in this study were obtained from the collected sausage casing and crawfish samples. To obtain the blank samples, a portion of each collected sample was tested for the presence of the target metabolites of NF using the developed LC-Q-Orbitrap HRMS method. The samples that showed no detectable levels of the target analytes were then designated as blank samples for their respective matrices. These blank samples were subsequently used for the preparation of matrix-matched calibration standards and quality control samples during the method validation process. By sourcing the blank samples from the same pool of collected samples, we ensured that the blank matrices closely resembled the actual samples in terms of their composition and potential matrix effects.

### 2.4 Washing procedure steps

The tissue washing procedure followed a protocol established by the European Reference Laboratory (EURL) for residues of veterinary drugs and contaminants in food of animal origin [4]. A 1.0 ± 0.02 g homogenized portion is weighed into a 50 mL polypropylene tube and subjected to a series of four solvent washes. The first wash involves adding 6 mL of a 50:50 MeOH/water solution (v/v) to the sample. This is followed by shaking at 700 rpm for 7.5 min and centrifugation at 4 °C for 10 min. The supernatant is discarded, and the sample proceeds to the next washing step. The second wash involves adding 6 mL of a 75:25 MeOH/water solution (v/v) to the sample, followed by the same shaking and centrifugation protocol as the first wash. Again, the supernatant is discarded, and the sample proceeds to the next step. The third wash consists of adding 6 mL of pure MeOH, followed by the same shaking and centrifugation protocol as the first and second washes. The supernatant is discarded, and the sample proceeds to the final washing step which involves adding 2 mL of water, followed by shaking for 20 s and centrifugation using the same parameters as the previous steps. The supernatant is discarded, and the sample is ready for analytical preparation as previously described for total residues.

### 2.5 Sample preparation and derivatization

After the washing steps, 4.0 mL of water was added to each tube, followed by 0.5 mL of 1 M HCl. Next, 150 µL of 2-NBA was added to the mixture. The tubes were mechanically shaken for 30 s at 700 rpm and then incubated for four hours at 60 °C while protected from light. Finally, 5 mL of 0.1 M KH_2_PO_4_ was added, followed by 300 µL of 1 M NaOH to achieve a pH of 7.00 ± 0.5. The tubes were then shaken briefly.

### 2.6 Liquid liquid extraction (LLE) procedure

**Fig. 1:**
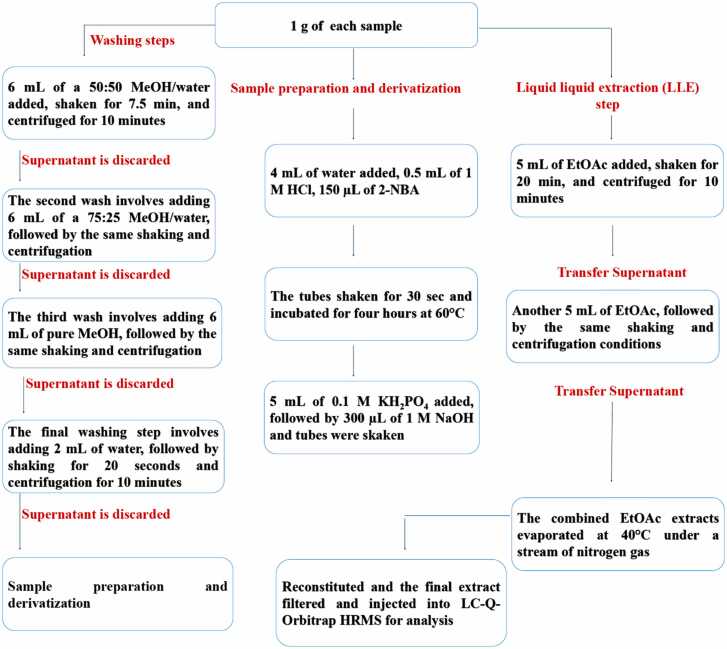
Flowchart of the analytical procedures for nitrofuran (NF) metabolites in sausage casing and crawfish, involving washing, derivatization, and LLE steps, coupled with LC-Q-Orbitrap HRMS.

Following neutralization, the solution undergoes LLE by adding 5 mL of EtOAc. The biphasic mixture is then shaken at 700 rpm for 20 min. Subsequently, the mixture is centrifuged at 4 °C for 10 min to enhance phase separation, with the denser aqueous layer settling to the bottom and the lighter EtOAc layer, containing the NF metabolites, rising to the top. The supernatant is then transferred into a 15 mL polypropylene tube for collection. The neutralized solution is subjected to a second round of LLE by adding another 5 mL aliquot of EtOAc, followed by shaking and centrifugation under the same conditions. The supernatant is carefully transferred into the same 15 mL polypropylene tube used in the previous extraction step. This process yields a combined EtOAc extract with a total volume of 10 mL. The EtOAc extract is evaporated at a temperature of 40 °C under a stream of nitrogen gas until nearly all the solvent is removed, resulting in an oily residue. This concentrate is then reconstituted in a 1 mL from dissolving solution and the mixture is subjected to sonication. The reconstituted sample is subsequently transferred to a new 15 mL centrifuge tube and centrifuged at 4500 rpm for 10 min at 4 °C. Following centrifugation, the reconstituted solution was then filtered through a 0.45 μm porous poly tetrafluoroethylene (PTFE) membrane filter into an amber glass vial, rendering it suitable for analysis. The sample was then ready for injection into the HPLC Orbitrap HRMS for further analysis as summarized in Fig. 1.

### 2.7 Chromatographic conditions

The chromatographic separation process was performed utilizing a Thermo Scientific Vanquish High Performance Liquid Chromatography (HPLC) system, manufactured by (Thermo Scientific in Bremen, Germany). The separation of analytes was accomplished by employing a ZORBAX Eclipse Plus C18 reversed-phase column (150 mm × 4.6 mm, 5 μm). The mobile phase consisted of eluent A (aqueous solution containing 2 mM ammonium acetate) and eluent B (ACN with 0.1 % formic acid). The chromatographic separation was performed using a gradient elution method with a total run time of 10 min. Initially, the mobile phase composition was set to 5 % eluent B, which was maintained for 0.3 min. Over the next 5 min, the proportion of eluent B was linearly increased to reach 100 %. This composition was held for 3 min before returning to the starting conditions of 5 % eluent B within a 2-minute re-equilibration period. The mobile phase was delivered at a constant flow rate of 0.5 mL/min. The column temperature was set at 40 °C to ensure consistent chromatographic performance, while the sample tray was maintained at 25 °C. For each analysis, a sample volume of 30 µL was injected into the chromatographic system.

### 2.8 Mass spectrometry conditions

Mass spectrometric analysis was conducted employing a Q-Exactive Orbitrap mass spectrometer (Thermo Scientific, Bremen, Germany), which was equipped with a heated electrospray ionization (HESI) source operating in positive ionization mode. The HESI source temperature was maintained at 350 °C, while the capillary temperature was set to 325 °C. An electrospray voltage of 3.70 kV was applied to facilitate ionization. The S-lens RF level was optimized to 50 volts to enhance ion transmission efficiency. Sheath and auxiliary gas flows were precisely regulated to 50 and 12 arbitrary units, respectively, to ensure stable and reproducible ionization conditions. The automatic gain control (AGC) target, which determines the number of ions accumulated in the Orbitrap analyzer, was set to 3.10^6^, while the maximum injection time (IT), which represents the maximum time allowed for ion accumulation, was set to 100 ms. These parameters were optimized to achieve optimal signal-to-noise ratios and mass accuracy. Full scan data acquisition was performed in positive ion mode at a high mass resolution of 70,000 FWHM (full width at half maximum), which enables accurate mass determination and facilitates compound identification. The mass range for full scan acquisition was set from *m/z* 100–500, covering a wide range of biologically relevant molecules. The instrument was operated in a Full MS/vDIA (variable Data Independent Acquisition) scan type, with a resolution of 70,000 for Full MS scans and 17,500 for vDIA scans. This advanced acquisition mode allows for comprehensive and unbiased data acquisition, enabling the identification and quantification of a range of analytes in complex biological samples. Data acquisition and processing were carried out using TraceFinder software (version 4.1, Thermo Fisher Scientific, Bremen, Germany).

### 2.9 Method validation

The validation of the analytical method was conducted in strict accordance with the EU Commission Implementing Regulation (CIR) 2021/808 [34]. The validation process entailed a comprehensive evaluation of critical parameters, including linearity, repeatability, reproducibility, recovery, decision limit (CCα), detection capability (CCβ), limit of detection (LOD), and limit of quantification (LOQ). Linearity, which refers to the ability of the analytical method to generate test results that exhibit a direct proportional relationship with the concentration of the analyte in the sample within a specified range, was extensively assessed in both the solvent and matrix. Calibration curves were constructed at five concentration levels, and clear linearity was rigorously tested across a concentration range ranging from 0.25 to 5 μg/L. Precision, which encompasses both repeatability and within-laboratory reproducibility, was validated using different blank samples of sausage casings and crawfish, obtained from different sources, and analyzed over three days to ensure robustness. These samples were verified to be devoid of the target analytes before use. The testing levels included three concentrations (0.25, 0.5, and 0.75 μg/kg), and for each concentration, six replicates (n = 6) were analyzed on the same day using matrix-matched calibration curves. This process was repeated over three different days, introducing variations in time, operator, and the calibration status of the LC-HRMS/MS equipment to ensure the robustness and reproducibility of the method. Recovery, also known as trueness, is a crucial parameter in analytical method validation that quantifies the proportion of an analyte successfully retrieved at the conclusion of an analytical procedure compared to the initial quantity of the analyte present in the original sample. This parameter serves as a measure of the method's accuracy. A high recovery percentage suggests that the method is capable of accurately measuring the analyte concentration, while a low recovery may indicate the presence of matrix effects or other factors that hinder the accurate quantification of the analyte. The CCα represents the threshold value above which a sample can be deemed non-compliant, with an associated error probability of α. Conversely, the value of 1 – α signifies the statistical confidence, expressed as a percentage, that the established threshold has been surpassed. CCβ is defined as the lowest quantity of the analyte that can be detected or quantified with a likelihood of error represented by β. In accordance with the guidelines set forth by the European Commission [34], for analytes with no specified Maximum Residue Limits (MRLs), The equations used to calculate CCα and CCβ for unauthorized NF metabolite compounds are as follows:

CCα = LCL + k(one-sided, 99%) × (combined) standard measurement uncertainty at LCL

CCβ = STC + k(one-sided, 95%) × (combined) standard measurement uncertainty at or above the STC

In these equations, LCL stands for the lowest concentration level, STC stands for the screening target concentration, which is the concentration level at which the analytical method is designed to detect the presence of the analyte with a specified level of confidence. A k-factor of 2.33 shall be used to CCα, and 1.64 shall be used for CCβ. Furthermore, the LOD, which represents the lowest amount of an analyte in a sample that can be detected but not necessarily quantified, and the LOQ, which is the concentration level at which the analyte can be reliably detected and measured with a specified degree of accuracy and precision, were determined. LOD is calculated as three times the standard deviation of the lowest concentration divided by the slope of the calibration curve, while LOQ is calculated as ten times the same standard deviation divided by the slope. Relative matrix effect or matrix factor (MF) experiments were conducted to assess the extent of ion suppression or enhancement caused by the sample matrix. The MF was quantified as described Khaled et al. [35] by comparing the slope of matrix-matched calibration curves (Slope M) with the slope of calibration curves prepared in MeOH solvent (Slope S) using the following equation:

ME% = 100 × ((Slope M)/(Slope S)) - 1

This equation provides a measure of the matrix's influence on the analyte's analysis. A result of 100 % indicates the absence of MF, meaning that the sample matrix does not interfere with the analyte's signal. Results above 100 % suggest ion enhancement, where the matrix components amplify the analyte's signal, leading to higher signal intensity. Conversely, results below 100 % indicate ion suppression, where the matrix components reduce the analyte's signal, resulting in lower signal intensity.

### 2.10 Proficiency testing (PT)

To guarantee the accuracy of the validated method, two proficiency testing (PT) samples from the Food Analysis Performance Assessment Scheme (FAPAS) were analyzed. The specific sample rounds investigated, namely 02475 and 02502, focused on NF metabolite analytes relevant to the study. PT is a valuable tool for assessing the performance of analytical methods and ensuring their suitability for the intended purpose. By participating in these FAPAS rounds and analyzing the PT samples, The accuracy and reliability of the analytical methods were evaluated against established standards. The results obtained from the analysis of the PT samples were compared to the assigned values provided by FAPAS, allowing for an independent assessment of the method's performance.

## 3 Results and discussion

### 3.1 Optimization of the liquid chromatography conditions

**Fig. 2:**
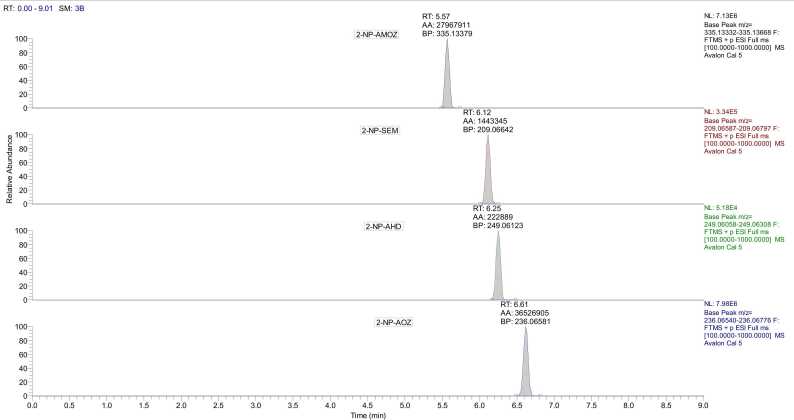
Representative chromatograms demonstrating the successful separation of NF metabolites at a concentration of 0.5 μg/L.

The chromatographic separation was carried out using a ZORBAX Eclipse Plus C18 (150 mm × 4.6 mm, 5 μm). This stationary phase proved to be highly effective in improving the peak shapes and reducing the retention times of the NF metabolites under investigation. The superior performance of the ZORBAX column is clearly evident in Fig. 2, which showcases the enhanced chromatographic separation achieved. These results are consistent with the findings reported in earlier studies [27], [36], [37], [38]. Further validating the suitability of this stationary phase for the analysis of the target compounds. In the aqueous phase, both ammonium acetate and ammonium formate were evaluated, with ammonium acetate showing a more pronounced increase in mass spectrometry sensitivity compared to ammonium formate. Consequently, ammonium acetate was selected as the preferred salt for the ensuing experiments. Subsequently, the influence of ammonium acetate concentration on the system was investigated by testing a range of concentrations from 0.1 to 2 mM. It was observed that elevated concentrations of ammonium acetate led to a marked improvement in peak shape for all NF metabolites. Based on these findings, an optimal concentration of 2 mM ammonium acetate was chosen for the remainder of the study. The outcomes of this investigation align with the conclusions drawn from prior research on this topic [2], [32], [38], [39]. In the organic phase in our optimization process, we compared the effects of ACN and MeOH as assessing their impact on sensitivity. The infusion experiments revealed that employing ACN led to a substantial enhancement in signal intensity for all NF metabolites compared to the results achieved when using MeOH. To enhance and stabilize the ionization process, the addition of acids to the mobile phase was explored [30]. We integrated 0.1 % formic acid with ACN to enhance the ionization efficiency and sensitivity of analytes accepted for positive ionization. The methodical inclusion of formic acid is aligned with our goal of optimizing the detection capabilities of our HPLC-Orbitrap HRMS system.

### 3.2 Optimization of the preparation procedure

#### 3.2.1 Liquid liquid extraction (LLE)

Extracting analytes from an animal food matrix while minimizing interference and maximizing recovery poses significant challenges and represents the most critical and demanding step in the analytical process [6]. The use of a polar organic solvent such as EtOAc proves to be effective in terms of enhancing analytical recovery and obtaining a cleaner extract, thereby optimizing the extraction process and improving the overall quality of the analytical results. In this study, 10 mL of EtOAc was employed to achieve high recovery of the analytes from the sample matrix. The extraction procedure was performed in two sequential steps to maximize the recovery efficiency of the analytes. This dual-stage approach was employed to ensure exhaustive extraction and to optimize the overall recovery of the target compounds from the sample matrix.

#### 3.2.2 Optimization of the derivatization

**Fig. 3:**
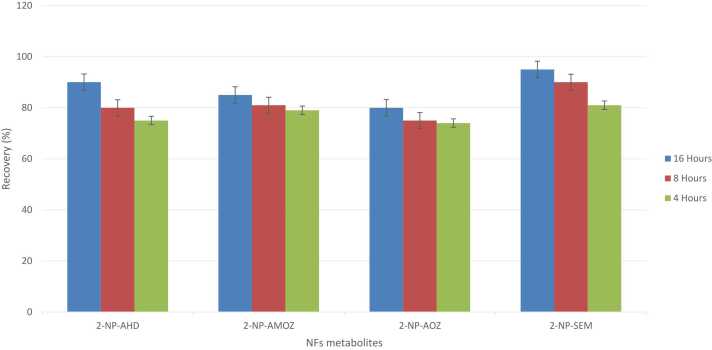
Comparison of derivatization times for NF metabolites: 16 h at 37°C, 8 h at 45°C, and 4 h at 55°C.

NF metabolites are characterized by their relatively low molecular weight (MW) and exhibit high solubility in polar solvents. Furthermore, they readily undergo ionization, which presents significant challenges for their direct analysis using LC-MS/MS techniques [1]. Hence derivatization of the samples is conducted. The process of derivatization offers several advantages: (i) it increases the MW of the side chain, (ii) it enhances the sensitivity of mass spectrometry detection by shifting the monitored ions to a region with reduced background noise, and (iii) it improves the specificity of the fragment ions, facilitating more accurate identification of the analytes [8]. In this study, we employed derivatization of the NFs metabolites using 2- NBA. We conducted trials by incubating the samples at varying temperatures and durations: 16 h at 37 °C, 8 h at 45 °C, and 4 h at 55 °C. As illustrated in Fig. 3, the recovery rates for all NF metabolites did not exhibit significant differences across the tested conditions.

### 3.3 Optimization of Q-Orbitrap HRMS parameters

The analysis was carried out using a Q-Exactive Orbitrap mass spectrometer (MS) in Full MS/vDIA scanning mode, operating in positive ion mode. The initial full mass scan served dual purposes: firstly, it facilitated the screening and quantification of target analytes, and secondly, it allowed for retrospective investigation of unknown compounds. Confirmation of the target analyte's identity was achieved through the generation of fragment ions, which played a pivotal role in the identification process. Upon detection of a target analyte with a signal intensity exceeding the predetermined threshold, all ions within the specified *m/z* range were allowed to pass through the quadrupole and enter the C-trap. The ions were then transferred to the higher-energy collision dissociation (HCD) cell for fragmentation without prior selection, as per the FullMS/vDIA mode [40]. This fragmentation process was essential for generating characteristic fragment ions, which aided in the unambiguous identification of the target compound. The fragments generated in the collision cell were collected in the C-trap and then transferred to the Orbitrap mass analyzer for further analysis, as reported by Jia et al. [41]. This analytical workflow provides a comprehensive examination of the compounds, beginning with a full MS scan and followed by a sequence of data-independent scans that focus on fragment ions generated using specific fragmentation energies. Table 1 presents a detailed summary of the four NF metabolites, including their compound names, precise precursor masses, fragment ions, and molecular formulas. This information is crucial for the accurate identification and characterization of the target compounds. The mass deviation of all diagnostic ions was consistently below 5 ppm for the entire mass range, ensuring accurate mass measurements and reliable identification of the target analytes. The effective resolution of the Q-Orbitrap HRMS system was selected to be fit for purpose and exceeded 70,000 at FWHM for the entire mass range. Full scan spectra were recorded for the identification of NF metabolites, and diagnostic ions with a relative intensity of more than 10 % in the reference spectrum of the calibration standards and matrix-matched standards were selected, including the molecular ion (when present at ≥ 10 % intensity of the base peak) and characteristic fragment ions. The selected fragment and product ions were diagnostic for the NF metabolites measured, and non-selective transitions were omitted whenever possible. The signal-to-noise (S/N) ratio of all diagnostic ions was greater than or equal to 3:1.

**Table 1:** Retention times, molecular formulas, and mass spectrometry parameters of the four targeted nitrofuran metabolites.

Analyte	Retention time	Molecular formula	Adduct	Precursor *m/z*	Product ion *m/z*	Ionization mode
2-NP-AHD	6.25	C_10_H_8_N_4_O_4_	[M + H]^+^	249.06183	134.02352, 114.02971, 108.04425,	Positive
2-NP-AMOZ	5.57	C_15_H_18_N_4_O_5_	[M + H]^+^	335.13500	291.14480, 262.11834, 128.10686, 100.07555, 86.060040	Positive
2-NP-AOZ	6.61	C_10_H_9_N_3_O_4_	[M + H]^+^	236.06658	149.03450, 134.02354, 101.03448, 92.025670, 78.046400	Positive
2-NP-SEM	6.12	C_8_H_8_N_4_O_3_	[M + H]^+^	209.06692	192.04015, 166.06112, 149.03446, 91.041650, 78.033740	Positive

### 3.4 Method validation

#### 3.4.1 Identification criteria

As per the requirements outlined in CIR 2021/808 [34], all validation parameters were found to be in compliance. During the development, validation, and analysis of real samples, the positive identification and confirmation of the analyte were based on a set of predefined criteria. These criteria included the retention time (RT), precursor ion, and product ion, all of which had to meet the established standards for the analyte to be considered positively identified and confirmed. The RT of AOZ, AMOZ, AHD, and SEM in the sample extracts were compared to those of the corresponding analytes in the calibration standards, matrix-matched standards, and matrix-fortified standards. Analysis revealed that the RT of the analytes in the sample extracts were within ±0.01 min of the RT of the respective standards. Furthermore, the RT of all analytes was found to be greater than twice the RT corresponding to the void volume of the column. Details of the theoretical RTs, average actual RTs, and RTs deltas are summarized in Table S1. Adherence to these criteria ensured the reliability and accuracy of the analytical method throughout the entire process. The presence of at least three product ions with a mass tolerance of less than 5 ppm and the RT were used as criteria for positive identification, as shown in Table 1.

#### 3.4.2 Selectivity of analytes

**Fig. 4:**
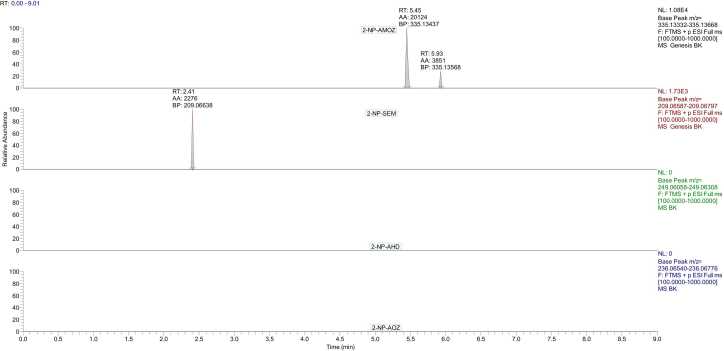
Chromatogram of the sausage casing blank sample.

Selectivity is a crucial validation parameter that ensures the accurate identification and quantification of analytes in the presence of potential interferences. The selectivity of the analytical method was evaluated by analyzing blank samples. The method demonstrated satisfactory selectivity, as no interfering peaks were observed at the retention times of the target analytes in the blank samples as shown Fig. 4. Moreover, the presence of potential interfering substances did not significantly affect the identification or quantification of the target analytes. The selective detection and confirmation of analytes were based on the comparison of retention times, precursor ions, and product ions with those of reference standards.

#### 3.4.3 Trueness and precision

The recovery rates for the NF metabolites in sausage casing matrix were found to be within the acceptable range of 77–109 %, with coefficients of variation (CVs) values for both repeatability and reproducibility not exceeding 15 %. Similarly, in the crawfish matrix, the recovery rates ranged from 86 % to 105 %, and the CVs remained below 15 %. These results underscore the high degree of accuracy exhibited by the analytical method across different food matrices as illustrated in Table 2. The data indicate that the analytical method used for determining the metabolites of NF in sausage casing and crawfish samples demonstrates acceptable accuracy (recovery rate) and precision (repeatability and reproducibility). The achievement of satisfactory recovery rates and low CVs in both sausage casings and crawfish matrices highlights the method's potential for wide applicability in the analysis of various food products.

**Table 2:** Recovery, coefficient of variation (CV) for repeatability, and reproducibility of the four targeted nitrofuran metabolites in sausage casing and crawfish samples.

	Sausage casing				Crawfish		
Analyte	Added concentration (μg/kg)	Recovery (%)	CV,repeatability (%)	CV,reproducibility (%)	Recovery (%)	CV,repeatability (%)	CV,reproducibility (%)
2-NP-AHD	0.25	78	10	12	86	9	11
	0.5	87	6	9	93	7	9
	0.75	97	8	13	87	8	9
2-NP-AMOZ	0.25	86	12	15	105	6	8
	0.5	109	7	8	98	7	8
	0.75	108	7	10	96	7	9
2-NP-AOZ	0.25	93	9	13	90	5	8
	0.5	84	10	13	93	6	7
	0.75	95	14	15	92	7	9
2-NP-SEM	0.25	91	10	11	104	10	11
	0.5	77	7	8	98	13	15
	0.75	88	8	12	97	9	13

#### 3.4.4 Linearity, limits of detection (LODs), and limits of quantification (LOQs)

The matrix-matched standard calibration curves demonstrated excellent linearity across a wide range of concentrations for the target analytes within the studied matrices. The calibration curves were generated using five concentration levels (0.25, 0.5, 1, 2, and 5 μg/L), as presented in Table 3. The correlation coefficients (R) for both matrix-matched standard calibration curves surpassed 0.9978, as illustrated in Fig. S1, indicating a strong linear relationship between the analyte concentration and the instrument response within the tested range (0.25–5 µg/kg). In the sausage casing samples, the LOD and LOQ ranged from 0.0218 to 0.0424 μg/kg and 0.0719–0.1390 μg/kg, respectively. For the crawfish samples, the LOD and LOQ values fell within the ranges of 0.0359–0.0596 μg/kg and 0.1184–0.1966 μg/kg, respectively. Among the four metabolites in sausage casing, AMOZ has the lowest LOD and LOQ. Similarly, in crawfish, AHD has the lowest LOD and LOQ, compared to the other metabolites. These low LOD and LOQ values highlight the method's high sensitivity and its ability to detect and quantify trace levels of NF metabolites in complex food matrices as shown Table 3. The combination of excellent linearity, strong correlation coefficients, and low detection and quantification limits underscores the method's suitability for accurate and reliable determination of nitrofuran metabolites in both sausage casings and crawfish samples.

**Table 3:** Linearity, limits of detection (LOD), and limits of quantification (LOQ) of the four targeted nitrofuran metabolites in sausage casing and crawfish samples.

Analyte	Sausage casing				Crawfish			
	R	Range (μg/kg)	LOD (μg/kg)	LOQ (μg/kg)	R	Range (μg/kg)	LOD (μg/kg)	LOQ (μg/kg)
2-NP-AHD	0.9992	0.25–5	0.0252	0.0831	0.9998	0.25–5	0.0359	0.1184
2-NP-AMOZ	0.9987	0.25–5	0.0218	0.0719	0.9978	0.25–5	0.0459	0.1514
2-NP-AOZ	0.9995	0.25–5	0.0424	0.1390	0.9984	0.25–5	0.0596	0.1966
2-NP-SEM	0.9998	0.25–5	0.0421	0.1380	0.9991	0.25–5	0.0369	0.1217

#### 3.4.5 The decision limit (CCα) and detection capability (CCβ)

In this study, CCα and CCβ values were evaluated for each target compound, and the results are summarized in Table 4. For sausage casing samples, the CCα values ranged from 0.31 to 0.39 μg/kg, while the CCβ values fell within the range of 0.29–0.35 μg/kg. Similarly, for crawfish samples, the CCα values varied from 0.30 to 0.34 μg/kg, and the CCβ values were found to be between 0.29 and 0.31 μg/kg. In both sausage casing and crawfish samples, AMOZ and AHD consistently had the lowest levels of CCα, with AOZ showing the highest CCα levels in sausage casing and SEM having the highest CCα in crawfish. For CCβ, AMOZ had the lowest in sausage casings, while AHD and AOZ had the lowest in crawfish. Conversely, AOZ showed the highest CCβ levels in sausage casing, and SEM had the highest CCβ in crawfish. The determination of CCα and CCβ is crucial in assessing the performance of analytical methods, particularly in the context of regulatory compliance.

**Table 4:** Decision limit (CCα), detection capability (CCβ), and relative matrix effect (MF) for the four targeted nitrofuran metabolites in sausage casing and crawfish samples.

Analyte	Sausage casing			Crawfish		
	CCα (μg/kg)	CCβ (μg/kg)	MF (%)	CCα (μg/kg)	CCβ (μg/kg)	MF (%)
2-NP-AHD	0.33	0.31	−14	0.30	0.29	−9
2-NP-AMOZ	0.31	0.29	−11	0.33	0.30	−11
2-NP-AOZ	0.39	0.35	−17	0.31	0.29	−7
2-NP-SEM	0.35	0.32	−8	0.34	0.31	−6

#### 3.4.6 Relative matrix effect (MF)

The MF was evaluated for both sausage casing and crawfish matrices, and the results are presented in Table 4. In the sausage casing matrix, a suppression effect was observed, ranging from −17 % to −8 %. AOZ has the highest signal suppression at −17 %, while SEM has the lowest at −8 %. Similarly, the crawfish matrix also exhibited a suppression effect, with values ranging from −11 % to −6 %. AMOZ and AOZ have the highest signal suppression at −11 %, while SEM has the lowest at −6 %. The MF is a common phenomenon in LC-MS/MS analysis, where the presence of co-eluting matrix components can influence the ionization efficiency of the target analytes. The suppression effect observed in both matrices highlights the importance of employing matrix-matched calibration curves to compensate for the matrix-induced signal suppression.

### 3.5 Comparing our findings to those of previous studies analyzing NF metabolites in food

In accordance with section 1.2.4.2 of the Commission Implementing Regulation (CIR) EU 2021/808 [34], a system of identification points was used to select adequate acquisition modes and evaluation criteria. As NF metabolites are considered unauthorized or prohibited substances, a minimum of 5 identification points is required for confirmation. In our method, chromatographic separation using LC provided 1 identification point. The use of HRMS with the Q-Orbitrap instrument allowed for the acquisition of full scan ions and HRMS product ions, contributing 1.5 and 2.5 identification points. The combination of LC separation, full scan HRMS ion, and multiple HRMS product ions for each precursor yielded a total of 5 identification points, meeting the minimum requirement for confirming the identity of NF metabolites in the tested matrices. To assess the performance of the developed LC-Q-Orbitrap HRMS method, a comparative analysis was conducted against previous studies focusing on the determination of NF metabolites in various food matrices [30], [31], [33], [42], [43]. The key parameters used for the comparison included the instrument, extraction method, samples, column, mobile phase, CCα, CCβ, and recovery as summarized in Table 5. Our method demonstrated superior sensitivity, with lower LOD and LOQ values compared to the alternative techniques reported in the literature. The improved sensitivity of our method highlights its effectiveness in accurately detecting and quantifying NF metabolites across a range of food matrices, making it a promising tool for future applications in food safety and monitoring.

**Table 5:** Comparative analysis of the developed LC-Q-Orbitrap HRMS/MS method and alternative techniques for the determination of nitrofuran metabolites in various food matrices.

Analyte	Instrument/Extraction method	Samples	Column	Mobile phase	CCα (μg/kg)	CCβ (μg/kg)	Recovery (%)	Reference
AOZ, AMOZ, AHD, SEM	UHPLC–QqQ-MS/MS / 10 mL of 0.2 M of HCl, derivatization with 100 μL of 0.1 M 2-NBA, 1.5 mL of 0.1 M KH_2_PO_4_, 0.2 mL of 2.5 M NaOH. Then SPE using Oasis HLB, eluted with 6 mL EtOAc and evaporated.	Seafood	Kinetex C18 column (50 mm×2.1 mm, 2.6 μm)	10 mM ammonium formate/MeOH	1.5–2.6	1.6–2.2	73–100	Valera-Tarifa et al., 2013 [31]
AOZ, AMOZ, AHD, SEM	HPLC-FLD/ washed using normal saline (10 mL, 0.9 %) and EtOAc (10 mL). 5 mL 1 % HCL, derivatization with DAOC, microwaving then injected	Bread, Instant noodle, pork, chicken, shrimps, prawn	Eclipse XDB-C18 column (4.6 mm × 150 mm, 5 μm)	5 % ACN containing 0.1 % FA/0.1 % FA in ACN	LOD= 0.20–0.30 μg/kg	LOQ= 0.70–1.00 μg/kg	93.8–98	Yu et al., 2019 [43]
AOZ, AMOZ, AHD, SEM	HPLC-FLD/ washed using normal saline (10 mL, 0.9 %) and EtOAc (10 mL). 5 mL 1 % HCL, derivatization with DAOC, microwaved then injected	Marine products	Eclipse XDB-C18 column	5 % ACN containing 0.1 % FA/ACN	LOD= 0.22–0.29	LOQ= 0.69–0.95	91.39–104.93	Luo et al., 2019 [30]
AOZ	Immunosensor/label-free electrochemical impedimetric immunosensor	Pork, shrimps, honey, carp, swine casing, and eel muscle	-	-	LOD= 60 ng/mL	-	91.4–106.2	Yang et al., 2011 [42]
AOZ, AMOZ, AHD, SEM	UPLCMS/MS / 10 mL of 2 M HCL, derivatization with 2-NBA 0.3 mL (0.05 mol L^−1^ solution in DMSO), cooled, 500 μL trisodium phosphate, NaOH, SPE using Oasis HLB, finally eluted with 6 mL EtOAc and injected	Aquatic products	Acclaim® RSLC C18 column (100 mm × 2.1 mm, 2.2 μm)	5 mM ammonium acetate 0.1 % acetic acid/ACN	LOD= 0.5	LOQ= 1.5	88–112	Zhang et al., 2016 [33]
AOZ, AMOZ, AHD, SEM	LC-Q-Orbitrap HRMS/ 4 mL water, 0.5 mL of 1 M HCl, derivatization with 2-NPA, 5.0 mL of 0.1 M KH_2_PO_4,_ 300 µL of 1.0 M NaOH to adjust pH 7. 10 mL EtOAc, evaporated under stream nitrogen gas then injected	Sausage casing and crawfish	ZORBAX Eclipse Plus C18 reversed-phase column (150 mm × 4.6 mm, 5 μm)	aqueous solution containing 2 mM ammonium acetate/ACN with 0.1 % FA	CCα= 0.30–0.39 LOD= 0.0218–0.0596	CCβ = 0.29–0.35 LOQ= 0.0719–0.1966	77–109	This study

### 3.6 NF Metabolites stability in post-extraction autosampler storage

To ensure the integrity and reliability of the analytical results, we conducted a comprehensive study to evaluate the stability of NF metabolite residues in the final extract when stored in glass vials in an autosampler at + 25°C over varying time periods. The stability assessment was performed by monitoring the stability of each compound at 12, 24, and 48 h post-extraction. As shown in Fig. S2, after 12 h of storage, degradation ranging from 1 % to 10 % was observed for each compound, indicating a relatively stable period for the final extract. However, the stability of the NFs significantly decreased after 24 h of storage. The degradation rates continued to increase at the 48-hour mark, with compounds losing 17–25 % of their initial concentrations. These findings underscore the importance of analyzing samples within 24 h of extraction to ensure accurate and reliable results.

### 3.7 Real samples analysis

The developed method was applied to determine the presence of NF metabolite residues in 35 samples, including 30 crawfish samples and 5 sausage casing samples. These samples were collected from various local markets across Egypt. Out of 30 crawfish samples, a total of 5 tested positive for the presence of SEM and AHD, resulting in an overall detection rate of 16.66 as shown in Table 6. SEM was detected in the range of 0.039–0.5 μg/kg, with residues present in slightly more than one-sixth of the crawfish samples. In contrast, AHD was detected in only one crawfish sample, at a concentration of 0.2 μg/kg, resulting in a detection frequency of 3.33 %. On the other hand, sausage casing samples did not show any positive detections for nitrofuran metabolite residues. These findings are consistent with the results reported in previous studies on various food matrices and honey [37], [44], [45], [46]. Moreover, the results align with the trends observed in a recent study analyzing RASFF notifications for fish between 2002 and 2022 [16].

**Table 6:** Residues of nitrofuran metabolites in sausage casing and crawfish samples collected from Egyptian retail markets.

Commodity	Sample number	Detected samples	Analyte	Range (μg/kg)		Mean (μg/kg)	Frequency (%)
				Minimum	Maximum		
Crawfish	30	5	2-NP-SEM	0.0390	0.5	0.17	16.67
			2-NP-AHD	0.2	-	-	3.33
Sausage casing	5	0	-	-	-	-	**-**

### 3.8 Proficiency testing (PT)

As part of the Food Analysis Performance Assessment Scheme (FAPAS), PT samples from rounds 02475 and 02502 were analyzed in fish samples to validate the performance and calculation methodology of the optimized assay. In round 02475, the method slightly underestimated the concentrations of AHD and AOZ, with Z-scores of −0.9 and −0.3, respectively. Neither analyte was detected in round 02502. This is consistent with the assigned values, as AMOZ and SEM were also not detected in round 02475. However, AMOZ and SEM concentrations were successfully quantified in round 02502, with Z-scores of −0.5 and −0.4, respectively (Table 7). The method demonstrated satisfactory performance in both FAPAS rounds as all reported Z-scores fall within the acceptable range. The Z-score is a statistical measure of the laboratory's accuracy that represents the deviation of the laboratory's result from the assigned value, expressed in terms of standard deviations. A Z-score between −2 and + 2 is generally considered satisfactory as specified by the FAPAS reports. This indicates that the optimized assay's performance and calculation methodology are reliable and consistent with the expected values established by the proficiency testing scheme.

**Table 7:** Proficiency testing (PT) outcomes for fish analysis using the validated method in the Food Analysis Performance Assessment Scheme (FAPAS).

FAPAS round	Analyte	Assigned value (μg/kg)	Found (μg/kg)	Z-score	Observations
02475	2-NP-AHD	2.70	2.59	−0.9	Satisfactory
	2-NP-AOZ	1.50	1.43	−0.3	Satisfactory
	2-NP-AMOZ	0	Not detected	-	Satisfactory
	2-NP-SEM	0	Not detected	-	Satisfactory
02502	2-NP-AMOZ	3.42	3.29	−0.5	Satisfactory
	2-NP-SEM	1.25	1.18	−0.4	Satisfactory
	2-NP-AHD	0	Not detected	-	Satisfactory
	2-NP-AOZ	0	Not detected	-	Satisfactory

## 4 Conclusions

The study presents a highly reliable and efficient method for the quantification of four NF metabolites in sausage casing and crawfish using LC-Q-Orbitrap HRMS. The developed method employs a simplified sample pre-treatment approach based on acid hydrolysis, derivatization, and LLE. This approach significantly reduces analysis time and increases sample throughput. The Full MS/vDIA scanning mode, coupled with accurate masses of parent and fragment ions, ensures high method sensitivity and the ability to determine residues in the range of 0.25–5 μg/kg. The validation procedure included selectivity, linearity, LOD, LOQ, trueness, repeatability, reproducibility, CCα, and CCβ. All validation parameter values were found to meet the intended use and established criteria, demonstrating the reliability of the developed method. The successful application of the method was demonstrated through the analysis of two proficiency testing (PT) samples and real samples, identifying 5 positive samples among 35 tested. The results highlight the effectiveness of the developed method in providing an integrated strategy for the screening and quantification of NF metabolite residues in food matrices. Continuous monitoring studies should be conducted regularly to determine the presence of veterinary drug residues in aquaculture products and food of animal origin, identify their sources, and implement secure preventive and remedial strategies.

## CRediT authorship contribution statement

**Lamia Ryad:** Writing – review & editing, Visualization, Resources, Methodology, Investigation, Data curation. **Omar Khaled:** Writing – review & editing, Writing – original draft, Visualization, Validation, Methodology, Investigation, Formal analysis, Data curation. **Fawzy Eissa:** Writing – review & editing, Visualization, Methodology, Investigation, Conceptualization. **Nermine Gad:** Writing – review & editing, Visualization, Resources, Methodology, Investigation, Data curation.

## Declaration of Competing Interest

The authors declare that they have no known competing financial interests or personal relationships that could have appeared to influence the work reported in this paper.

## Data Availability

Data will be made available on request.
