# Supplementary material for: Determination of nitrofuran metabolites in sausage casings and crawfish using LC-Q-Orbitrap HRMS: Method development and validation
Source: Toxicol Rep. 2025 Jun 19;15:102076. doi: 10.1016/j.toxrep.2025.102076 (PMC12256301; doi:10.1016/j.toxrep.2025.102076)
Supplement: Table S1 — Supplementary material [file mmc1.docx]

| Analyte | Theoretical RT (min) | Average actual RT (min) | Average RT delta (min) |
| --- | --- | --- | --- |
| 2-NP-AHD | 6.25 | 6.24 | -0.01 |
| 2-NP-AMOZ | 5.57 | 5.57 | 0.00 |
| 2-NP-AOZ | 6.61 | 6.62 | 0.01 |
| 2-NP-SEM | 6.12 | 6.13 | 0.01 |

Table S1: Representative batch samples from the validation of crawfish analysis, presenting the theoretical retention time (RT), average actual RT, and average RT delta, for each analyte.

**Fig. S1: Calibration curve and peak of NF metabolites in solvent at concentrations ranging from 0.25 to 5 µg/L.**

**Fig. S2: Stability assessment of NF metabolites in the final extract stored in glass vials over various time intervals (12, 24, and 48 hours), indicating the degradation percentage for each compound at the specified time points.**
